# Microbiota: A potential orchestrator of antidiabetic therapy

**DOI:** 10.3389/fendo.2023.973624

**Published:** 2023-01-27

**Authors:** Bingyao Liu, Linlin Zhang, Hang Yang, Hongting Zheng, Xiaoyu Liao

**Affiliations:** Department of Endocrinology, Chongqing Education Commission Key Laboratory of Diabetic Translational Research, the Second Affiliated Hospital of Army Medical University, Chongqing, China

**Keywords:** gut microbiota, metabolites, type 2 diabetes, antidiabetic therapy, glucoregulatory agents

## Abstract

The gut microbiota, as a ‘new organ’ of humans, has been identified to affect many biological processes, including immunity, inflammatory response, gut-brain neural circuits, and energy metabolism. Profound dysbiosis of the gut microbiome could change the metabolic pattern, aggravate systemic inflammation and insulin resistance, and exacerbate metabolic disturbance and the progression of type 2 diabetes (T2D). The aim of this review is to focus on the potential roles and functional mechanisms of gut microbiota in the antidiabetic therapy. In general, antidiabetic drugs (α-glucosidase inhibitor, biguanides, incretin-based agents, and traditional Chinese medicine) induce the alteration of microbial diversity and composition, and the levels of bacterial component and derived metabolites, such as lipopolysaccharide (LPS), short chain fatty acids (SCFAs), bile acids and indoles. The altered microbial metabolites are involved in the regulation of gut barrier, inflammation response, insulin resistance and glucose homeostasis. Furthermore, we summarize the new strategies for antidiabetic treatment based on microbial regulation, such as pro/prebiotics administration and fecal microbiota transplantation, and discuss the need for more basic and clinical researches to evaluate the feasibility and efficacy of the new therapies for diabetes.

## 1 Introduction

The gut microbiota is the largest human microecosystem, and is a complex community consisting of more than 500 microbial species (1, 2). The gut bacteria are mainly composed of five phyla, namely, *Firmicutes*, *Bacteroidetes*, *Actinobacteria*, *Proteobacteria* and *Verrucomicrobia*, and more than 90% of the bacteria belong to *Firmicutes* and *Bacteroidetes* (3). Dysbiosis of the gut microbiota is associated with the occurrence and development of various diseases, such as immunological diseases (4, 5), inflamed intestinal diseases (6, 7), mental disorders (8, 9), and metabolic diseases (10–13). The homeostasis of gut microbiota could be affected by host genetics (14, 15), environmental factors (16), medicines (17, 18), and lifestyles (19). Kawano et al. found that high-fat, high-sugar diet aggravated glucose intolerance and insulin resistance in mice by depleting Th17 inducing microbes, particularly segmented filamentous bacteria (SFB) (20). Besides, one randomized-controlled trial on the effects of non-nutritive sweeteners (NNS) in human found that sucralose and saccharin supplementation significantly impaired glycemic response in healthy adults. Transplantation of microbiomes from top and bottom NNS responders into germ-free (GF) mice indicated that the alteration of gut microbiota induced by NNS could cause personalized glycemic alterations, as exemplified by sucralose (21). In the context of metabolic disorders, the gut microbiota is closely connected with the dysfunction of glucose and lipid metabolism, inflammation response and insulin resistance (22–24). Recent studies found that GF mice are protected against high-fat diet (HFD) induced glucose intolerance and obesity (25). In addition, colonization of GF mice with gut microbiota isolated from conventionally raised obese donors led to a significant increase in insulin resistance and body fat content (26, 27). Consequently, the gut microbiota was identified to be closely linked to the pathogenesis of diabetes and obesity.

Recently, numerous studies have shown that the gut microbiota is changed in both type 1 diabetes (T1D) and type 2 diabetes (T2D) patients, which indicates an etiological relationship between the gut microbiota and diabetes. The microbiome of T1D presented a decrease in diversity, an increase in the *Firmicutes* and *Firmicutes*/*Bacteroidetes* ratio, and a reduction in *Proteobacteria* and *Bacteroidetes* (28–30). Compared with controls, 16S rRNA data of individuals with T1D showed a lower proportion of butyrate-producing and mucin-degrading bacteria (28), and fecal microbiota transplantation (FMT) from healthy donors halted the decline in endogenous insulin production in new-onset T1D patients (31). Similarly, in metagenome-wide association studies, T2D patients were characterized by a moderate degree of gut microbial dysbiosis, an increase in various opportunistic pathogens and a decrease in the abundance of some universal butyrate-producing bacteria, such as *Clostridium* species (12, 32). Among the commonly and consistently reported findings, the genera of *Bifidobacterium*, *Bacteroides*, *Faecalibacterium*, *Akkermansia* and *Roseburia* were negatively associated with T2D, while the genera of *Ruminococcus*, *Fusobacterium*, and *Blautia* were positively associated with T2D (33). Moreover, it has been found that the altered gut microbiota in diabetes is associated with metabolic parameters. For instance, the ratio of *Bacteroidetes* to *Firmicutes* and the ratios of *Bacteroides*-*Prevotella* group to *C. coccoides*-*E. rectale* group were correlated significantly and positively with plasma glucose concentration (34). These studies suggest that dysbiosis of gut microbiota might greatly contribute to the occurrence and progression of diabetes.

In this review, we focus on the potential roles and mechanisms of gut microbiota in the antidiabetic therapy. Briefly, we summarize the contribution of microbiota to glucoregulatory therapy for T2D, and the demonstrated mechanism of microbial metabolites in regulating inflammation response, insulin resistance and glucose homeostasis, and discuss the new strategies for targeting intestinal bacteria in the treatment of diabetes.

## 2 Contribution of microbiota to glucoregulatory therapy for T2D

Recently, it has been reported that the alteration of gut microbiota mediated by antidiabetic agents was involved in the regulation of glucose and lipid metabolism in diabetes. In this part, we concluded the role of commonly used glucoregulatory agents in the regulation of the composition and function of gut microbiota.

### 2.1 α-glucosidase inhibitor

α-glucosidase inhibitor, as one fermentation product of bacterial strains derived from *Actinoplanes* sp. *SE50* (35), inhibits α-glucosidase activity and slows carbohydrate uptake in the brush border of the small intestinal mucosa to reduce postprandial hyperglycemia (36, 37). This results in an increase in dietary carbohydrate in the distal intestine, where it becomes food for the gut bacterial community. Some studies have focused on the effect of α-glucosidase inhibitors on the gut microbial composition in diabetes. Acarbose, voglibose, and miglitol are pseudo-carbohydrates that competitively inhibit α-glucosidase activity. Acarbose is the most used glucoregulatory drug of this family. In T2D patients and rats, acarbose treatment significantly increased the content of *Bifidobacterium* (38–40), which has an anti-inflammatory effect, and has been reported to decrease in diabetes (41). The increased *B. longum* showed a positive correlation with the level of high-density lipoprotein cholesterol (HDL-C), a potent protective factor against atherosclerosis (38). One controlled crossover trial involving 52 Chinese prediabetic patients discovered that short chain fatty acids (SCFAs)-producing taxa, such as *Lactobacillus, Faecalibacterium* and *Prevotella*, were greatly increased after acarbose treatment (42). SCFAs, including acetate, propionate and butyrate, are fermented from indigestible dietary carbohydrates by intestinal bacteria. It has been demonstrated that SCFAs have important roles in stimulating glucagon-like peptide-1 (GLP-1) and insulin secretion, ameliorating inflammation response, and improving glucose intolerance and insulin resistance (43–45). Besides, some SCFAs-producing bacteria, such as *Lactobacillus* strains, have a broader spectrum of inhibitory activity than acarbose, effectively inhibiting α-glucosidase and β-glucosidase activities (46), and have beneficial effects on insulin resistance and weight loss (47, 48). Moreover, administration of acarbose to treatment-naïve T2D patients also changed the relative level of microbial genes involved in bile acid metabolism, and increased the ratio of primary bile acids to secondary bile acids. The correlation analysis demonstrated that patients with a higher baseline abundance of *Bacteroides* had lower levels of secondary bile acids, and exhibit more beneficial therapeutic responses to acarbose treatment (49). That is, the acarbose-changed microbiome was associated with its treatment efficiency for T2D. Additionally, voglibose, another α-glucosidase inhibitor, decreased the ratio of *Firmicutes* to *Bacteroidetes*, and increased the circulating levels of taurocholic and cholic acid, which ultimately has systemic effects on body weight and lipid metabolism in HFD mice (50).

### 2.2 Biguanides

Among biguanides, metformin is recommended by the American Association of Clinical Endocrinologists (AACE) as a first-line hypoglycemic treatment of T2D for its glucose lowering and insulin sensitizing effects (51), and is the most commonly used oral glucoregulatory agent. The hypoglycemic effect is ascribed to suppress hepatic gluconeogenesis, improve the uptake and utilization of glucose in peripheral tissues, promote the release of glucagon-like peptide-1 (GLP-1) and peptide YY (PYY) from L cells, and enhance insulin sensitivity (52–55). In T2D patients and murine models, metformin has been identified to modify gut microbial structure and diversity. Indeed, Shin et al. found that metformin-treated HFD mice showed significant differences in the abundance of *Firmicutes* and *Bacteroidetes*, when compared with non-treated mice (56). Similarly, Lee and Ryan also observed that metformin induced an increase in the abundance of *Bacteroidetes* and *Verrucomicrobia*, and a decrease in the abundance of *Firmicutes* in HFD mice (57) (58). At the genus level, the SCFAs-producing bacteria such as *Blautia*, *Bacteroides*, *Butyricoccus* and *Phascolarctobacterium* were enriched by metformin treatment (59). Particularly, the abundance of *Akkermansia*, which are mucin-degrading bacteria, was significantly increased in metformin treated mice (57, 58). Administration of cultured *A. muciniphila* to HFD mice significantly enhanced glucose tolerance, and attenuated serum lipopolysaccharide (LPS) and inflammation status by inducing Foxp3 regulatory T cells in visceral adipose tissue (56).

Similar with the results observed in rodents, administration of metformin to T2D patients increased the relative abundances of *Lactobacillus* and *Akkermansia*, as well as the SCFAs-producing bacteria such as *Bifidobacterium*, *Prevotella*, *Megasphaera* and *Butyrivibrio* (60). *B*. *adolescentis* has been shown to negatively correlate with HbA1c. Thus, this taxon might contribute to the glucose-lowering effect of metformin (61). Besides, metformin treatment decreased the relative abundance of *Intestinibacter*, and enriched *Escherichia* in human feces. Whereas *Escherichia* probably plays an important role in the side-effects of metformin (62). In addition to changing the microbial composition, metformin treatment also promoted a functional shift in the gut microbiome, increasing the levels of propionate, butyrate and bile acids in the gut of T2D patients (61–63). As reported by CT Jiang, metformin-increased glycoursodeoxycholic acid (GUDCA) was identified as an intestinal FXR antagonist that improved various metabolic endpoints in mice with established obesity (64). Even though the mechanism of hypoglycemia of metformin is not completely clear, its administration altered the gut microbial composition and function, which might be involved in the regulation of glucose metabolism.

### 2.3 Incretin-based glucoregulatory agents

Incretins are small peptide hormones secreted by intestinal endocrine cells responding to nutrient ingestion, mainly glucose and fat. There are two main incretin hormones in humans: GLP-1 and GIP (gastric inhibitory peptide). They stimulate insulin secretion in a glucose-dependent manner, regulate appetite and gastric emptying, and play a crucial role in the local gastrointestinal physiology (65). Additionally, PYY is one of the first hormones to be expressed in the developing fetal gastrointestinal tract. The primary role of PYY is acting as a potent anorectic hormone. PYY_3-36_, a major form of PYY in both the gut mucosal endocrine cells and the circulation, induces satiety by targeting the appetite-regulating system of the hypothalamus (66). Insulin-like peptide-5 (INSL5) is an orexigenic gut hormone, secreted by enteroendocrine L cells present in the ileum and colon together with GLP-1 and PYY. INSL5 modestly inhibited forskolin-stimulated GLP-1 secretion in NCI-H716 cells (67).

Naturally intact GLP-1 is degraded rapidly, mainly *via* enzymatic inactivity by dipeptidyl peptidase-4 (DPP-4). Clinically, incretin-based therapies for diabetes include GLP-1 receptor agonists (incretin mimetics) and DPP-4 inhibitors (DPP-4i, incretin enhancers). The GLP-1 receptor agonist, such as liraglutide, promotes the synthesis and secretion of insulin, and inhibits appetite, gastric emptying and food intake to control blood glucose level in diabetes (68, 69). In the liraglutide-treated obese and diabetic rodents, in addition to the obviously improved glucose and lipid metabolism, substantial rearrangements of bacterial structure were observed. It has been found that liraglutide decreased the obesity-related phylotypes (such as *Erysipelotrichaceae*, *Roseburia*, and *Parabacteroides*), and increased the lean-related phylotypes (such as *Prevotella*, *Lactobacillus* and *Akkermansia*) *(* 70, 71). The bacteria positively correlated with hepatic steatosis associated parameters were decreased in HFD rats upon liraglutide intervention (72). Colonization of GF mice with gut microbiota from liraglutide-treated diabetic mice improved glucose-induced insulin secretion and regulated the intestinal immune system (73). Additionally, some SCFAs-producing bacteria, including *Bacteroides*, *Lachnospiraceae* and *Bifidobacterium*, were selectively enriched in liraglutide-treated diabetic rats (74). The liraglutide treatment was also associated with bile acid metabolism (75). SCFAs stimulates GLP-1 expression and secretion through binding with G-coupled protein receptor GPR43 and GPR41 (76), while bile acids *via* other receptors such as FXR and TGR5 (77). However, one randomized placebo-controlled trial in adults with T2D suggested that the beneficial effects of liraglutide on glucose metabolism are not linked to the intestinal microbiota composition, when used as add-on therapies to metformin or sulphonylureas (78). As mentioned in the study, the limitations including the lack of dietary monitoring/standardization and control of co-medication use might be the reason for the inconsistency with the results observed in animal experiments.

DPP-4i is one of the most extensively used oral glucoregulatory agent, and its target DPP-4 enzyme has a high expression level in the small intestine (79, 80). DPP-4i exerts a hypoglycemic effect by inhibiting the degradation activity of DPP-4 to increase the blood level of GLP-1 and GIP (81). Recently, some subjects have revealed that the gut microbial community is modified by DPP-4i administration. In obese and diabetic rats, sitagliptin treatment moderately restored the microbial composition to that in controls. At the phylum level, sitagliptin reduced the ratio of *Firmicutes* to *Bacteroidetes* (82). At the genus level, probiotics *Bifidobacterium* and *Lactobacillus* were obviously increased after sitagliptin treatment (39), while the abundance of SCFAs-producing bacteria (*Roseburia* and *Clostridium*) was not significantly altered by sitagliptin. Consistent with these findings, in our previous study, we found that DPP-4i (sitagliptin and saxagliptin) altered the gut microbial composition in T2D patients and HFD mice, especially reducing the ratio of *Firmicutes* to *Bacteroidetes*, and fecal microbiota transfer from sitagliptin-treated T2D patients to GF mice improved the glucose tolerance in recipients (83). Moreover, we have demonstrated that sitagliptin also changed the pattern of fecal and serum metabolites in HFD mice and T2D patients, including the levels of succinate, branched-chain amino acids and aromatic amino acids (83, 84). We are still exploring the molecular mechanism by which DPP-4i improves glucose homeostasis through gut microbiota-derived metabolites, especially indole derivatives.

### 2.4 Traditional Chinese medicine

Traditional Chinese medicine (TCM), generally also known as botanical medicine or phytomedicine, is an important scientific and technological resource with therapeutic or other human health benefits. Generally, the use of TCM herbal formula (FuFang in Chinese) is fundamental and includes several medicinal herbs. TCM has been reported to be an effective remedy for metabolic disorders, including T2D (85). In recent ten years, TCM with antidiabetic effects has also been identified to have effects on the structure of the gut microbiota (86, 87). Berberine is the major pharmacological component of the Chinese herb *Coptis chinensis* (Huanglian). The oral bioavailability of berberine is poor (below 5%), and over 95% stays in the gut (88, 89), which suggests a potential effect on the intestinal bacteria. Actually, it has been reported that berberine induced a marked shift in the structure of gut microbiota, some of which were selectively enriched in putative SCFAs-producing bacteria, including *Blautia*, *Allobaculum*, *Bacteriodes* and *Butyricoccus*, consequently leading to an increase in the fecal SCFAs concentration in obese and T2D rats (59, 90, 91). Berberine was also identified to promote the gene expression of ACK, MMD and BUT, which are the key enzymes in the synthetic pathway for SCFAs (92). In addition, berberine has been reported to have a hypoglycemic effect in T2D patients by inhibiting deoxycholic acid biotransformation by *Ruminococcus bromii* (89). Likewise, administration of Gegen Qinlian Decoction (GQD) to T2D rats decreased the levels of blood glucose and inflammatory cytokines, and altered the gut microbial composition, especially increasing the proportions of SCFAs-producing and anti-inflammatory bacteria, and decreasing the proportions of conditioned pathogenic bacteria associated with diabetic phenotype (93). Moreover, a double-blinded trial revealed a dose-dependent deviation of gut microbiota in response to GQD treatment in T2D patients, and the deviation occurred before significant improvement in T2D symptoms. The GQD enriched *F. prausnitzii* was positively correlated with β-cell function, and negatively correlated with FBG, HbA1c and postprandial blood glucose levels in T2D patients (94). In addition, a water extract of Ganoderma lucidum mycelium (WEGL), which is a medicinal mushroom used in TCM, decreased the ratio of *Firmicutes* to *Bacteroidetes* and endotoxin-bearing *Proteobacteria* levels, as well as improved inflammation response and insulin resistance in HFD mice. The effects were transmissible *via* FMT from WEGL-treated mice to HFD mice (95). Taken together, these studies indicate that the amelioration of hyperglycemia and insulin resistance by TCM in T2D is at least partially mediated by structural regulation of the gut microbiota.

## 3 Potential mechanisms of microbial effects on metabolism in diabetes

**Figure 1:**
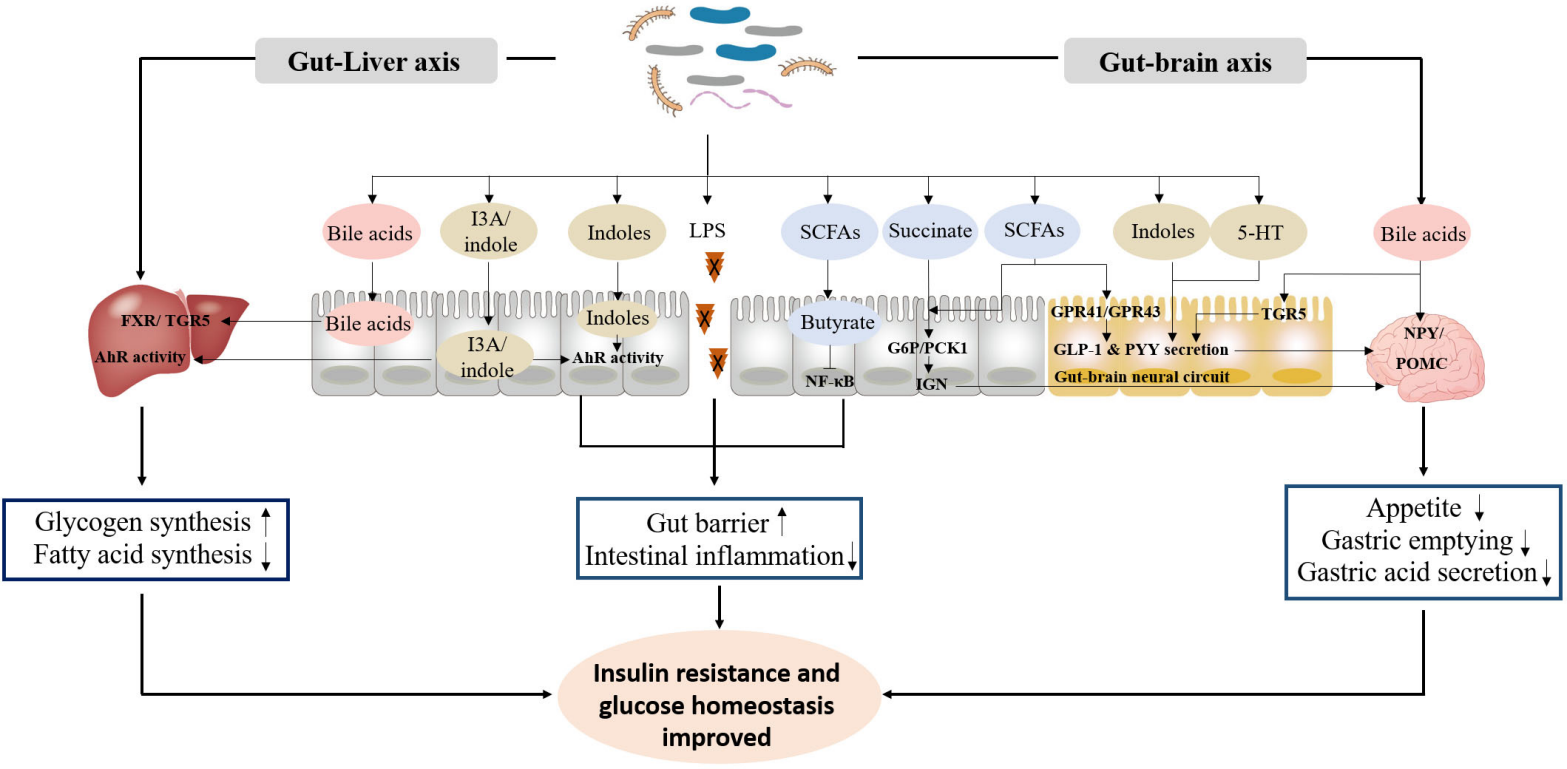
The primary functional mechanism of bacterial metabolites in the improvement of insulin resistance and glucose homeostasis. ① LPS, SCFAs and indoles improve gut barrier, and inhibit intestinal inflammation response. ② Bile acids, indoles, SCFAs, succinate and 5-HT promote GLP-1 and PYY secretion, and inhibit appetite, gastric emptying and gastric acid secretion through gut-brain axis. ③ Bile acids and indoles promote glycogen synthesis and insulin sensitivity, and inhibit fatty acid synthesis through gut-liver axis.

The exploration of the potential mechanisms of gut microbiota in the occurrence and progression of diabetes mainly focuses on microbial metabolites, especially LPS, SCFAs, bile acids, and indoles, which are involved in the regulation of gut barrier, inflammation response, insulin resistance and glucose homeostasis (96–98) (**Figure 1**).

### 3.1 Gut barrier and inflammation response

Dysbiosis of gut microbiota induces an increase in the levels of pathogens and endotoxins in the intestine. These toxic substances will induce intestinal macrophages to release proinflammatory factors such as TNF-α and histamine, and induce inflammatory response in intestinal cells, subsequently increasing the permeability of intestinal mucosa and damaging the intestinal barrier (99, 100). It has been recognized that low-grade inflammation is one of the most important pathophysiologic factors resulting in the progression of T2D (101). The intestinal bacteria produced LPS is a triggering factor for low-grade inflammation *via* the LPS-CD14 system, and further induces metabolic endotoxemia and insulin resistance (102). Two species (*B. vulgatus* and *B. dorei*) with potential benefits for T2D have been found to decrease gut microbial LPS production, and effectively suppress pro-inflammatory immune response in a mouse model (103). Similarly, administration of *A. muciniphila* to diabetic mice reduced the level of serum LPS, relieved intestinal inflammation, and improved glucose homeostasis (104). The butyrate-producing bacteria (such as *Roseburia* and *Faecalibacterium*) is known to eliminate constitutive NF-κB and suppress its activation, subsequently reducing colonic paracellular permeability and preventing inflammatory response (105, 106).

In addition, indole, as the most prevalent metabolite of tryptophan, is produced by various bacterial species, including those belonging to the genera *Bacteroides*, *Escherichia*, and *Clostridium* (107). Recent studies have revealed that indoles-induced aryl hydrocarbon receptor (AhR) activation may be an important way for bacteria to improve inflammatory status and insulin resistance (108). For example, indole derivates indole-3-carboxaldehyde upregulates the production of anti-inflammatory factor IL-22 by activating AhR in intestinal immune cells (109, 110).

### 3.2 Gut-brain axis

Bacteria produced metabolites, such as SCFAs, bile acids and indoles, have effects on the intestinal endocrine cells to modulate the secretion of gut hormones, which play important roles in the gut-brain axis (111). SCFAs promote the secretion of GLP-1 and PYY by binding to membrane receptors GPR41 and GPR43 on endocrine L cells (112, 113). Likewise, depletion of Firmicutes and Bacteroidetes with antibiotics in diet-induced obesity mice increased production of gut-derived taurocholic acid, which promoted GLP-1 secretion from L-cells (114). Indole was also identified to promote GLP-1 secretion from L cells during short exposure, but reduce GLP-1 release over longer period. These different effects were due to whether indole enhance the acute secretion of GLP-1 by enhancing Ca^2+^ entry or reduce its release by blocking NADH dehydrogenase to slow ATP production (115). The secreted GLP-1 and PYY can inhibit appetite, gastric emptying and gastric acid secretion through acting on pro-opiomelanocortin (POMC) and neuropeptide Y (NPY) neurons in the hypothalamus (116). Similarly, bile acid reaches hypothalamus, specifically activates the expression of AgRP/NPY neuron membrane receptor TGR5, and then regulates the appetite of mice (117). Additionally, Gilles Mithieux and his colleagues reported that propionate, butyrate and succinate activated intestinal gluconeogenesis (IGN) to initiate a gut-brain neural circuit that has beneficial effects on glucose tolerance and insulin resistance (43, 118).

Additionally, gut-derived serotonin (5-hydroxytryptamine, 5-HT), as a metabolite of tryptophan, is released from enterochromaffin (EC) cells. 5-HT has been discovered to shaped the gut microbiota composition, and inhibited β-defensins production from colonic epithelial cells (119, 120). Besides, 5-HT signaling is reported to be involved in the increase of L-cell density in mouse and human intestine and elevation of GLP-1 secretory capacity (121). Moreover, the activation of GLP-1 leads to the release of 5-HT in brain, subsequently reducing appetite and body weight (122).

### 3.3 Gut-liver axis

Accumulating evidence demonstrated that bile acids as pleiotropic signaling molecules modulate specific host metabolic pathways and inflammatory response involving gut-liver crosstalk, *via* nuclear and G-Protein-coupled receptors, such as the farnesoid X-activated receptor (FXR) and TGR5 (64, 123). FXR signaling represents a core regulatory pathway involved in glucose and lipid metabolism, and maintains the barrier integrity and intestinal homeostasis (124, 125). TGR5 is a membrane receptor, mainly activated by lithocholic acid and deoxycholic, which are secondary bile acids produced by microbial metabolism. TGR5 has been discovered to have effects on inflammation, insulin pathway and glucose metabolism (126, 127).

Except for bile acids, tryptophan metabolite such as tryptamine and indole-3-acetate (I3A) attenuated inflammatory response and reduced fatty acid synthesis *via* activating receptor AhR in hepatocytes (128). SCFAs are readily absorbed by colonocytes upon produced. Butyrate is mainly utilized by colonic epithelium as an energy source, and a large amount of acetate enters systemic circulation, while propionate is primary utilized by the liver (129). Acetate and butyrate were found to be highly involved in liver palmitate and cholesterol synthesis, and propionate is a substrate for *de novo* gluconeogenesis in liver, not for lipogenesis (130).

In addition to gut-brain axis and gut-liver axis, microbial metabolites also play roles in adipose, pancreas and muscle, involved in the regulation of inflammatory response, insulin secretion, insulin sensitivity, etc. (44, 131, 132).

## 4 Targeting gut bacteria - a new therapeutic strategy for T2D

The molecular mechanism of gut microbiota in the pathophysiology of T2D is still not completely clear, while existing studies have indicated that some interventions, such as probiotics, prebiotics and FMT targeting gut bacteria improved glucose homeostasis and insulin resistance, which indicates that these interventions might be potential strategies for T2D therapy.

### 4.1 Probiotics and prebiotics

Probiotics, particularly *Lactobacilli* and *Bifidobacterium*, have recently emerged as prospective biotherapeutics in metabolic disease, which is supported by their established multifunctional roles in the prevention and treatment of metabolic disturbance (133, 134). In HFD mice, probiotics (*L. rhamnosus*, *L. acidophilus* and *B*. *bifidum*) treatment significantly reversed the impaired intestinal permeability, systemic inflammation and glucose intolerance (135). Administration of *L. plantarum* HAC01 to T2D mice obviously lowered blood glucose and HbA1c levels, and improved glucose tolerance and HOMA-IR. Notably, *L. plantarum* HAC01 increased the *Akkermansiaceae* family and increased SCFAs in serum (136). In randomized controlled trials, *L. acidophilus* and *B. lactis* or *B*. *bifidum* significantly ameliorated the FBG and antioxidant status in T2D patients (137, 138). Likewise, *L. paracasei* HII01 supplementation significantly decreased the levels of FBG, LPS, TNF-α, IL-6 and hsCRP in plasma of T2D patients compared with the placebo group (139).

Apart from *Lactobacillus* and *Bifidobacterium*, other bacteria associated with metabolic improvement may also be potential probiotics, such as *Akkermansia muciniphila*. Its abundance markedly increased after prebiotic feeding (140, 141), or metformin treatment in diabetic patients and mice (56, 61). *A. muciniphila Muc^T^* administration to diabetic mice increased the intestinal levels of endocannabinoids, which modulate inflammation, gut barrier and gut peptide secretion (104). Plovier et al. revealed that Amuc_1100, a specific protein isolated from the outer membrane of *A. muciniphila*, interacted with Toll-like receptor 2 (TLR2) to improve the gut barrier, and partly recapitulated the beneficial effects of the bacterium. Furthermore, administration with live or pasteurized *A. muciniphila* grown on synthetic medium is safe for obese individuals (142), which paves the way for its potential clinical application in metabolic syndromes. Another potential probiotic, *Parabacteroides goldsteinii*, was also identified to enhance intestinal integrity, improve inflammation and insulin resistance, and promote adipose tissue thermogenesis after oral administered to HFD mice (143).

Prebiotics are nondigestible carbohydrates, selectively stimulating the growth and activity of some bacteria, such as *Lactobacillus* and *Bifidobacterium* (144). Oligofructose-enriched diet reduced the ratio of *Firmicutes* to *Bacteroidetes*, increased the abundance of probiotics, such as *Bifidobacterium* and *Prevotella*, and improved glucose tolerance and L-cell function in ob/ob mice (145). Oligofructose also improved fasting blood glucose and glucose-stimulated insulin secretion, and reduced body weight gain in HFD mice, and the effects were modulated in a GLP-1 receptor-dependent manner (145, 146). Moreover, supplementation with oligofructose-enriched inulin to T1D participants for 12 weeks increased C-peptide, and improved intestinal permeability (147). However, some studies on consumption of pro/prebiotics by individuals with T2D have provided conflicting results, which do not provide evidence for the role of pro/prebiotics in the treatment of diabetes (148, 149). Therefore, the effects of pre/probiotics need to be confirmed by conducting more reliable clinical trials.

### 4.2 Fecal microbiota transplantation

FMT has been verified to be effective for the treatment of *Clostridium difficile* infection in immunocompromised patients (150) and subjects with depressive disorder (8). Intriguingly, FMT is also carried out in the treatment of metabolic syndrome, including diabetes and obesity. Transfer of fecal samples from metformin-treated T2D patients to GF mice showed that glucose tolerance was improved in recipient mice (61). Similarly, our study discovered that FMT from DPP-4i-treated T2D patients to GF mice also improved the glucose intolerance induced by HFD in recipient mice (83). Moreover, clinical studies showed that intestinal microbiota from lean donors to individuals with metabolic syndrome attenuated insulin resistance, increased gut microbial diversity, especially the levels of the butyrate-producing bacterium *Roseburia intestinalis*, and changed the plasma metabolites, such as γ-aminobutyric acid and tryptophan (151, 152). In addition, allogenic FMT using feces from metabolic syndrome donors decreased insulin sensitivity in metabolic syndrome recipients compared with using post-Roux-en-Y gastric bypass donors (153). However, we must emphasize that these studies did not report data on glucose levels during the intervention, and not all participants responded to FMT (152). The method is still very new and, thus, much more research must be dedicated to FMT, to explore the potential risks related to its impact on the physiological functions regulated by fecal microorganisms, most of which are unidentified and uncharacterized at present.

## 5 Conclusion

The gut microbiota is involved in the occurrence and development of diabetes, and responses to antidiabetic therapy both in intestinal tract and in extraintestinal tissues. In this review, we summarized the current studies about the effect and mechanism of gut microbiota in antidiabetic therapy, especially in improving inflammation, glucose homeostasis and insulin resistance modulated by antidiabetic agents. Although the molecular mechanism remains unclear, advances in sequencing technologies and bioinformatics have enabled the enormous complexity and number of metabolites derived from intestinal bacteria to be identified in biological samples. Current research suggests that LPS, SCFAs, bile acids and indoles are involved in regulating host metabolic homeostasis. However, which strains induce altered metabolites, and whether the strains can be cultured *in vitro* need to be investigated. Studies are also necessary to address where and how the altered metabolites regulate host metabolism. Given the many insights so far based on animal experiments, it will be key to determine which phenotypes observed in animal models are relevant to humans and could be repeated in humans. Therefore, more clinical trials should be summoned to clarify the potential therapeutic effect of gut bacteria and derived metabolites in diabetes.

## Author contributions

BL: consulted, analyzed and summarized the literatures, and drafted the manuscript. LZ consulted, analyzed and summarized the literatures. HY consulted the literatures. HZ: designed the study, and revised the manuscript. XL: designed the study, and drafted and revised the manuscript. All authors contributed to the article and approved the submitted version.
